# Determining the Factors Affecting Long-Term and Short-Term Survival of Breast Cancer Patients in Rafsanjan Using a Mixture Cure Model

**DOI:** 10.34172/jrhs.2021.51

**Published:** 2021-05-26

**Authors:** Sardar Jahani, Mina Hoseini, Rashed Pourhamidi, Mahshid Askari, Azam Moslemi

**Affiliations:** ^1^Department of Biostatistics, School of Medicine, Arak University of Medical Sciences, Arak, Iran; ^2^Department of Biostatistics, School of Public Health, Hamadan University of Medical Sciences, Hamadan, Iran; ^3^Department of Biostatistics and Epidemiology, School of Public Health, Kerman University of Medical Sciences, Kerman, Iran; ^4^Non Communicable Diseases Research Center, Bam University of Medical Sciences, Bam, Iran; ^5^Department of Biostatistics, School of Medicine, Arak University of Medical Sciences, Arak, Iran

**Keywords:** Breast cancer, Survival analysis, Cox model, Long-term survivors

## Abstract

**Background:** Breast cancer is one of the most common causes of death among women worldwide and the second leading cause of death among Iranian women. The incidence of this malignancy in Iran is 22 per 100,000 women. These patients have long-term survival time with advances in medical sciences. The present study aimed to identify the risk factors of breast cancer using Cox proportional hazard and Cox mixture cure models.

**Study design:** It is a retrospective cohort study.

**Methods:** In this cohort study, we recorded the survival time of 140 breast cancer patients referred to Ali Ibn Abitaleb Hospital in Rafsanjan, Iran, from 2001 to 2015. The Kaplan-Meier curve was plotted; moreover, two Cox proportional hazards and the Cox mixture cure models were fitted for the patients. Data analysis was performed using SAS 9.4 M5 software.

**Results:** The mean age of patients was reported as 47.12 ±12.48 years at the commencement of the study. Moreover, 83.57% of patients were censored. The stage of disease was a significant variable in Cox and the survival portion of Cox mixture cure models (P=0.001). The consumption of herbal tea, tumor size, duration of the last lactation, family history of cancer, and the type of treatment were significant variables in the cured proportion of the Cox mixture cure model (P=0.001).

**Conclusion:** The Cox mixture cure model is a flexible model which is able to distinguish between the long-term and short-term survival of breast cancer patients. For breast cancer patients, cure effective factors were the stage of the disease, consumption of herbal tea, tumor size, duration of the last lactation, family history, and the type of treatment.

## Introduction

Therapeutic advances in recent decades have reduced infectious diseases; nonetheless, the incidence of non-communicable diseases has increased significantly^1^. Cancer is responsible for 13% of all deaths worldwide and one of the most important concerns of health systems as the second leading cause of death from non-communicable diseases^2^. Breast cancer is one of the most commonly diagnosed cancers among women, accounting for 23% of all cancers. The incidence rate of this cancer in Iran is 22 per 100,000 women^3^. Although the prevalence of breast cancer in Iran is lower than that in the United States, Europe, and even Asia, it is expected that in the next 10 or 20 years, it will be double or triple close to the countries with high breast cancer rates ^4^.

Advances in medical science have led to long-term survival in cancer patients and even a cure in some cases ^5^. Survival analysis is used to analyze the expected duration of time until one event occurs. The time from an illness to death, the time to recovery from illness, the time to the onset or recurrence of the disease, the time for people to return to prison after the release are some examples of this analysis^6^. The most common method of data survival analysis is the Cox proportional hazards model, which assumes that if the duration of follow-up in the study is sufficient, the desired event will occur to all individuals ^7^. It has been observed that due to the advancement of medical science, some people in the study do not experience the desired event^5^. In a situation when the population of patients is heterogeneous, a part of patients have long-term survival or are completely cured, and another proportion of patients who do not respond to treatment have short-term survival. The use of ordinal survival analysis models is not appropriate when the study population is divided to two groups: people prone to the disease and those who are cured.

The cure models which are used in such cases have a better interpretation. They consider cure proportion in analysis and find the variables that affect the curing ^8^. These models are assigned to two categories of the mixture and non-mixture models. The mixture model is one of the most common cure models expanded by Boag in 1949 and the Berkson and Gage in 1952. Berkson and Gage (1952) developed the mixture cure fraction model in population-based cancer studies and divided the population into two groups: susceptible individuals to the event of interest and insusceptible individuals ^9^.

Subsequently, various studies presented and evaluated the parametric and semi-parametric mixture cure models. Nonetheless, the use of semi-parametric mixture cure models has received more attention ^7^. The present study used the Cox proportion hazards and the Cox mixture cure models to identify risk factors associated with breast cancer.

## Methods

 This retrospective cohort study was conducted on 140 breast cancer patients who were referred to Ali Ibn Abitaleb Hospital, Rafsanjan, Iran, from 2000 to 2015. The inclusion criterion was the diagnosis of breast cancer after 2000. Patient information was collected through medical records in the hospital's archives and pathology laboratory. Data were collected through face-to-face interviews with the patient or family members of deceased patients, as well as calling the patients who had migrated or were not available.

 In the present study, the time between the diagnosis of breast cancer and the time of patient's death due to this disease or the completion of the study was regarded as the response variable. Independent variables recorded for patients included the severity of the disease, the duration of the last breastfeeding, family history of cancer, consumption of herbal tea, tumor size, and surgical treatment, as well as such adjuvant methods as chemotherapy, radiotherapy, and hormone therapy. At the end of the study, survived patients were regarded as cured, and those with missing information after a certain period of follow-up were considered right-censored. As mentioned earlier, cure models are more appropriate for a heterogeneous population. A proportion of patients have long-term survival or are cured fully, and another section have short-term survival or do not respond to treatment. Cure models can be divided into two groups: mixture and non-mixture cure models. Mixture cure models assume that the population consists of two groups of cured and non-cured patients. The survival of cured patients exceeds that of non-cured ones. The mixture cure model is advantageous since it allows for separate covariate inference for cured and uncured patients.

 This model is written as follows:

 St|X=pX+1-pXS0 tX

 S (t | X) is a function of the survival of the whole population.

 X is a set of variables.

 p (X) is the ratio of cured patients.

 (1-p (X)) is a proportion of non-cured patients.

S_0_(t│X) is a function of survival of patients who are not cured.

 In the current study, a Kaplan-Meier plot was drawn, and two assumptions were tested, including the existence of a cure fraction in the study population and sufficient follow-up time for patients by Maller and Zhou tables. Moreover, the Cox proportional hazards and the mixture cure models were fitted. For the mixture cure model, the semiparametric Cox function in the short-term survival section and the logit link function in the long-term survival section were used due to simplicity and popularity. In addition, the Hazard ratio and Odds ratio are simple and useful indexes to interpret. Finally, it was calculated and the risk factors affecting cure were identified. Data were analyzed in SAS 9.4M5 software, and a p-value less than 0.05 was considered statistically significant.

## Results

The study included 143 patients (3 men and 140 women) with breast cancer in a 15 years period. Three men were excluded from the study. The mean age of the patients was 47.12±12.48 years. Out of 140 patients in the study, 23 (16.43%) cases died and 117 subjects (83.57%) survived. Table 1 presents the descriptive statistics of predictor variables measured for patients. The survival rates of 5 and 10 years with a confidence interval of 95% are reported in Table 2. The survival rate decreased during years.

**Table 1:** Demographic characteristics of patients with breast cancer

**Categorical variables**	**Number**	**Percent**
Type of treatment		
≤Two treatments ^a^	93	66.43
Surgery & chemotherapy & hormone therapy	15	10.71
Surgery& chemotherapy & radiation therapy & hormone therapy	32	22.86
Daily use of soft drinks		
Tea	61	43.57
Tea and other herbals	79	56.43
Stage		
2	94	67.14
3	35	25.00
4	11	7.86
Family history of cancer		
Yes	68	48.57
No	72	51.43
Marital status		
Married	138	98.57
Single	2	1.43
Smoking		
Yes	53	37.86
No	87	62.14
Caesarean section		
Yes	31	22.14
No	109	77.86
Abortion		
Yes	41	29.29
No	99	70.71
**Continuous variables**	**Mean**	**SD**
BMI	26.92	4.51
Age (year)	47.12	12.48
Tumor size (cm^3^)	17.39	20.64
The duration of last time breast-feed (month)	15.91	8.62

^a^ The patient received two treatments of three treatments contain Surgical, Chemotherapy and Hermione therapy.

**Table 2:** Survival rates of breast cancer patients with 95% confidence interval

**Time (yr )**	**Estimation of survival**	**SE**	**95% CI**
5	0.80	0.04	(0.72, 0.89)
7	0.76	0.05	(0.66, 0.86)
10	0.66	0.07	(0.54, 0.81)

**Figure 1:**
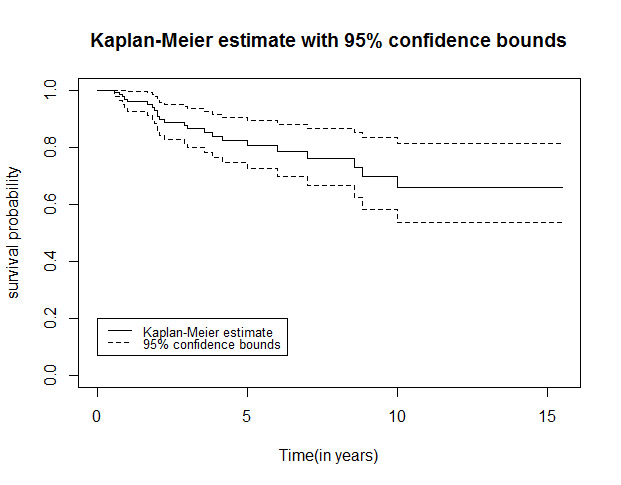
Kaplan-Meier plot for breast cancer patients

One common way to demonstrate the survived people in a study is to draw the Kaplan-Meier plot. Figure 1 displays the Kaplan-Meier plot with a 95% confidence interval. This survival curve appears to reach a plateau in 10 years after diagnosis. This indicates the presence of a sub-population in breast cancer patients who survives event-free by the end of the follow-up, clearly suggesting the appropriateness of a Cox mixture cure model.

Furthermore, the hypotheses of the cure fraction existence and the adequacy of the follow-up time of patients were accepted according to Maller and Zhou tables. In the present study, the mixture cure model and the Cox proportional hazards model were fitted, and the variables were selected using the backward stepwise elimination. Table 3 depicts the results of fitting the Cox proportional hazards model. In this model, the mortality risk has been reported based on the variables of treatment type and stage of cancer.

**Table 3:** Cox proportional hazards model results (AIC: 146.39)

**Variables**	**Estimated coefficient**	**SE**	***P*** **-value**	**HR**	**95% CI**
Type of treatment						
≤Two treatmentsa^a^	1.00					
Surgery & chemotherapy & hormone therapy	0.30	0.69	0.661	1.35	0.35	5.25
Surgery & chemotherapy & radiation therapy & hormone therapy	-0.90	0.61	0.132	0.40	0.12	1.32
**Stage**						
2	1.00					
3	3.28	0.81	0.001	26.77	5.60	127.80
4	4.97	0.88	0.001	144.94	25.70	816.24

^a^ The patient received two treatments of three treatments contain Surgical, Chemotherapy and Hermione therapy.

According to the results of Cox's model, the survival of patients in the three treatment groups did not differ significantly. Patients with stages 3 and 4 of the disease had a significantly higher hazard of death, compared to patients with stage 2 of cancer (Hazard ratios (HRs)) were 26.77 and 144.94, respectively). The results of the Cox mixture cure model are displayed in Table 4 in the presence of predictor variables and logit link function. In the long-term survival part, daily usage of infusions and a family history of cancer had a significant effect on the reduction of individuals' cure time. Moreover, one unit increase in tumor size and the duration of the last breastfeeding reduced the patients’ cure rate. Patients who received more than two treatments had a higher chance of cure in the long-term survival section and a higher risk of death in the short-term survival part. An increase in the degree of disease was associated with a decrease in patient survival in the short-term survival. Small sample size due to large HRs especially in the cured arm of the study.

**Table 4:** Results of the Cox mixture cure model with the Logit link function (AIC: 122.76)

**Variables**	**OR (95% CI)**	***P*** **-value**
Daily use of soft drinks		
No	1.00	
Yes	0.07 (4.07, 50.42)	0.001
Size of the tumor	0.76 (1.18, 1.45)	0.001
The duration of last time breast-feed (month)	0.93 (1.01, 1.16)	0.042
Family history of cancer		
No	1.00	
Yes	0.06 (4.05, 59.02)	0.001
Type of the treatment		
Two treatments ^a^	1.00	
Surgical treatment - Chemotherapy - Hermione therapy	8.13 (0.02, 0.72)	0.021
Surgery, Chemotherapy, Radiation Therapy, Hormone Therapy	0.50 (0.01, 0.13)	0.001
**Variables**	**HR (95% CI)**	***P*** **-value**
Stage		
2	1.00	
3	8.50 (1.86, 38.75)	0.005
4	33.31 (6.32, 175.31)	0.001
Type of treatment		
Two treatments*	1.00	
Surgical treatment - Chemotherapy - Hermione therapy	1.56 (0.39, 6.16)	0.521
Surgery, Chemotherapy, Radiation Therapy, Hormone Therapy	1.22 (0.34, 4.34)	0.753

^a^ Two treatments: Patient received two treatments of three treatments contain Surgical, Chemotherapy and Hermione therapy.

## Discussion

 In the present study, the survival rates of 5, 7, and 10 years were calculated with a 95% confidence interval for breast cancer patients, and the Kaplan-Meier survival curve was plotted. The existence of a cured fraction in the population is the basic assumption for different kinds of cure models (parametric and semiparametric, mixture, and non-mixture). Based on the obtained results, the Kaplan-Meier plot exhibited plateaus at the end of the curve. Thereafter, Cox proportional hazards model and the Cox mixture cure model were fitted, and the results were reported.

 In Cox proportional hazards model, the stage of disease was significantly associated with condensed survival time of patients. In the long-term survival of the cure model, the consumption of herbal tea, tumor size, duration of the last breastfeeding, and family history of cancer were associated with a significant reduction in odds of patients cure. The type of treatment for long-term survival was associated with an increased probability of recovery. A raise in the stage of disease was also linked to an increased hazard of death in the short term.

Survival-data analysis performs a crucial role in breast cancer research, considering the prediction of patient survival and its risk factors. The cure model is commonly used in cases where a proportion of patients have long-term survival. The cure model considers the ratio of cured people in survival analysis and provides a more accurate estimate of the survival fraction. In their study, Ghasemi et al. (2019) fitted two Weibull and Beta- Weibull Poisson non-mixture cure models to 270 women with breast cancer. In the stated study, the Beta- Weibull Poisson cure model had a better fit than the cure Weibull model.Some variables, including a tumor size of more than 5 cm in diameter and the third stage of cancer, showed significant relationships with a decrease in cure of patients ^10^. The results of the study by Ghasemi et al. are consistent with the findings of the present study.

Mohammadpour et al. examined the cure models in breast cancer patients in East and West Azerbaijan, Iran. In the Cox model, two variables of economic status and emergency hospitalization were identified as significant factors affecting the long-term survival of breast cancer patients ^11^. In addition, the 5-year survival rate of patients was estimated at 60.6% which is less than the values obtained in the present study.

In 2013, Asano et al. fitted the semi-parametric model of Cox and the cure model of Cox mixture with breast cancer patients. The results showed that the status of the hormone receptors, the disease grade, and the number of lymph nodes metastasized to the cure model were significantly. In both models, the grade of the disease and the metastatic status of the tumor affected patients' long-term survival ^12^. The results of the study by Asano et al. are in line with those obtained in the current research. In 2017, Hosseini et al. compared the Cox model, the cure model of the mixture log-normal, and the Weibull model. In the referred study, the most important factors affecting breast cancer were smoking, breast-feed, tumor size, and degree of disease.

According to the Akaike information criterion (AIC), the log-normal model was better than the Weibull model, and the results of the Cox model were closer to the clinical results ^13^. Hosseini et al. used parametric cure models, while we used a semiparametric mixture cure model. However, tumor size and degree of disease were significant variables in both studies. Among the notable limitations of this study, we can refer to the small sample size of breast cancer patients due to the small population of Rafsanjan, which resulted in obtaining large HRs, especially in the cured proportion of the study. It is proposed to use a large sample size to solve or reduce the problems of convergence in fitting cure models. Furthermore, urbanization is one of the most important factors contributing to the global epidemiology of breast cancer; therefore, it is recommended to involve urbanization in cure models.

## Conclusion

 The mixture cure model is more appropriate in studies with a proportion of cured patients since it considers the ratio of cured people in the estimation of survival fraction. This model is one of the most popular models used in medical science and breast cancer research since it is able to distinguish between the long-term and short-term survival of breast cancer patients.

## Conflict of interest

 The authors declare that they have no conflict of interest regarding the publication of the current study.

## Funding

 The present article was extracted from an MSc thesis and was financially supported by Arak University of Medical Sciences (IR.ARAKMU.REC.1398.285).

## Highlights

- The majority of breast cancer patients have long-term survival time.
- The cure model considers the ratio of cured people in survival analysis.
- The cure model provides a more accurate estimate of survival fraction, compared to the Cox model.
- Cox mixture cure is an effective model for the identification of the risk factors of breast cancer.
